# Testing for Testamentary Capacity in the Older Adult: A Model of Ethical Considerations for the Clinical Neuropsychologist

**DOI:** 10.3389/fpsyg.2019.01905

**Published:** 2019-08-22

**Authors:** Anne I. Roche

**Affiliations:** Department of Psychological and Brain Sciences, The University of Iowa, Iowa City, IA, United States

**Keywords:** older adult, testamentary capacity, ethics, ethical decision-making, clinical neuropsychology, assessment

## Abstract

The proportion of the United States population comprised of older adults is consistently growing. Older adults are often involved in making decisions regarding transfer of wealth, and cases involving questions of testamentary capacity are common. Neuropsychologists are well-positioned to perform evaluations of testamentary capacity given their knowledge and expertise surrounding assessment of cognitive and psychological functioning, as well as of neurodegenerative disease related to the aging process. Performing evaluations of testamentary capacity with older adults often comes with complex ethical considerations, however, and neuropsychologists could benefit from a decision-making model to aid in the organization of these multifaceted issues at the clinical-legal interface. The current paper proposes the implementation of Behnke’s “four bin” model to aid in the exploration of these complex ethical considerations and provides examples of how the model may be applied through two hypothetical case vignettes.

## Introduction

The proportion of the population consisting of older adults is consistently growing, as demonstrated by the percentage of United States residents age 65 and older accounting for 12.4% of the total population in year 2000 and 15.2% in 2016 (United States Census Bureau, 2017) and estimates indicating that they will account for approximately 21% of the population by 2030 and 24% of the population by 2060 (Mather et al., 2015). Many older adults are involved in decisions surrounding monetary transfer through the development and adjustment of their wills. In fact, older adults are currently involved in likely the most massive transfer of wealth in history (American Bar Association Commission on Law Aging and American Psychological Association, 2008; Brenkel et al., 2018; Hoffman, 2018). It is anticipated that the aging population will bring with it an increasing number of legal cases involving questions of testamentary capacity, which is already “the most frequently litigated form of capacity” (American Bar Association Commission on Law Aging and American Psychological Association, 2008, p. 16; Hoffman, 2018). At the most basic level, the legal construct of testamentary capacity can be defined as “a person’s ability to make or change a will” (Hoffman, 2018, p. 214).

Though decisions regarding testamentary capacity are ultimately determined in a legal context, healthcare professionals are often consulted to provide evaluations of testamentary capacity. Assessment can be requested by an individual and/or a lawyer to establish capacity in anticipation of a potential will-contest, by others who question the individual’s current capacity, or retrospectively after an individual has passed when challenging an existing will (Brenkel et al., 2018; Voskou et al., 2018). Clinical neuropsychologists may be in an optimal position to perform these evaluations given their knowledge surrounding the aging process, cognitive and functional assessment, decision-making capacity, neurodegenerative disease, and emotional/psychiatric disorders. Given this, neuropsychologists of course need to consider the most appropriate methods for assessment of capacity.

Broadly, previous work has suggested a functional approach to the evaluation of capacity (Sousa et al., 2014). A variety of writings have provided considerations for testamentary capacity assessment, suggesting utilization of cognitive testing in domains such as: attention, abstract and logical reasoning, information processing, verbal abstraction and comprehension, language abilities, semantic and autobiographical memory (including assessments encompassing both recall and recognition), executive functions, and decision-making capacity, while additionally emphasizing the importance of ecologically valid assessments (Moberg and Kniele, 2006; American Bar Association Commission on Law Aging and American Psychological Association, 2008; Sousa et al., 2014; Hoffman, 2018; Voskou et al., 2018). In addition to assessment of multiple cognitive domains, previous writings also highlight the importance of the assessment of psychiatric, personality, and emotional factors (American Bar Association Commission on Law Aging and American Psychological Association, 2008; Sousa et al., 2014; Voskou et al., 2018). Finally, previous work suggests value in using both self-report and performance-based functional assessments in order to gain insight into an individual’s ability to perform basic and instrumental activities of daily living (Sousa et al., 2014). Though previous work provides a useful framework for performing these assessments, there is still no one specific standardized assessment or assessment battery that is used to establish testamentary capacity (Brenkel et al., 2018). Given this lack of standardization, it becomes increasingly important that neuropsychologists are aware of the *ethical considerations* surrounding the evaluation of testamentary capacity when performing these evaluations.

Testamentary capacity evaluations have the potential to involve multiple ethical quandaries for the neuropsychologist. These ethical considerations include, but are not limited to, issues surrounding professional competency, informed consent, assessment selection and administration, multiple relationships, payment, autonomy and self-determination, and beneficence and non-maleficence. Though the literature to date has certainly acknowledged the importance of ethics in the evaluation of capacity in older adults with mental disorders (including dementia) (Katona et al., 2009) and in the evaluation of testamentary capacity specifically (Moberg and Kniele, 2006; Hoffman, 2018), clinicians faced with potential ethical dilemmas when performing testamentary capacity evaluations could benefit from the implementation of an ethical decision-making model to aid in the exploration of all relevant aspects of the specific situation and context that may influence ethical decision-making processes and outcomes. Additionally, the literature to date is lacking in its presentation of specific and detailed examples of ethical dilemmas that may arise specifically within testamentary capacity assessment. Thus, the aim of this article is to present how Behnke’s “four-bin” approach to ethics consultation can be applied within the context of testamentary capacity evaluation through the presentation of two case vignettes.

## Behnke’s “Four-Bin” Model

**FIGURE 1:**
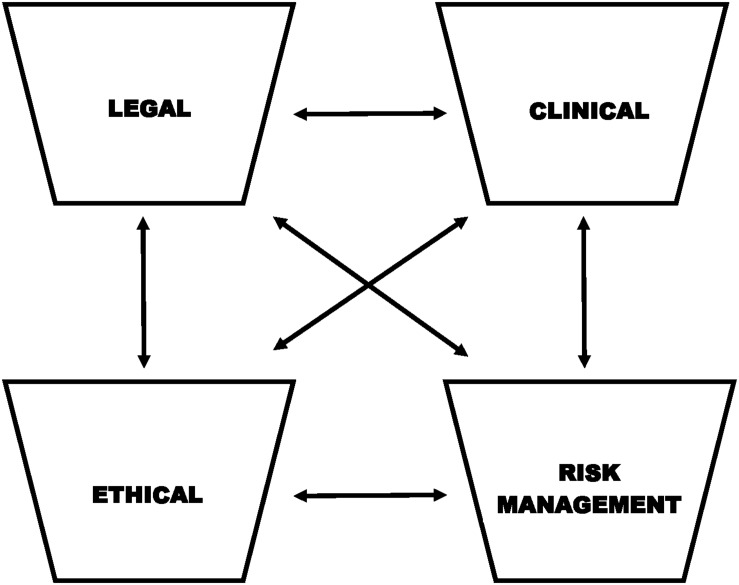
Adapted from Behnke’s “four-bin” model. Considerations include the differentiation and integration of: *legal* (federal and state law), *clinical* (assessment and treatment interests of the client), *ethical* (American Psychological Association Principles of Psychologists and Code of Conduct), and *risk management* (potential liability issues) issues and factors.

Behnke (2014) proposed a “four-bin” approach to ethics consultation in clinical practice settings. The four-bin model helps to differentiate and organize the issues, factors, and questions involved in an ethical dilemma in a manner that allows for the clinician to move forward in addressing the issues and coming to a decision. The four bins include: legal, clinical, ethical, and risk management (Figure 1). The *legal* bin includes issues associated with federal and state law. The *clinical* bin involves assessment and treatment interests of the client. The *ethical* bin involves considerations linked to the American Psychological Association’s (APA) Ethical Principles of Psychologists and Code of Conduct (APA Ethics Code; American Psychological Association, 2017). Finally, the *risk management* bin involves the exploration of liability issues that may arise subsequent to possible courses of action. Behnke emphasizes that the four bins are both different and interrelated, and most ethical dilemmas involve the integration of more than one, if not all, bin(s). Even still, the model provides a useful framework for organizing multiple perspectives and considerations when faced with a potential ethical dilemma within clinical practice. Two hypothetical case vignettes are presented below to provide examples of how Behnke’s model may be applied in these cases.

## Case Vignette #1

Ms. X is a 72-year old, recently widowed, Egyptian-American woman. Ms. X has lived in the United States for most of her adult life. She strongly identifies with Egyptian culture and Islamic religious practice. Ms. X’s husband passed away 9 months ago, and Ms. X reports continued significant struggles with grief; her primary care physician recently provided a formal diagnosis of major depressive disorder. Additionally, Ms. X had a previous neuropsychological evaluation approximately 15 months ago, at which point she was given the diagnosis of mild cognitive impairment (MCI) with a specific deficit in the domain of memory.

Ms. X’s husband had been the primary breadwinner and financial decision-maker in the household. Since his passing, Ms. X has decided that she would like to make some adjustments to her will, specifically in order to add some distant family members and her mosque as partial recipients of her bounty (inheritance). Ms. X’s lawyer has suggested that Ms. X complete an evaluation for testamentary capacity before adjusting her will. Specifically, Ms. X has reported that her children are encouraging her *not* to make any changes to her current will, and Ms. X’s lawyer would like to avoid litigation surrounding testamentary capacity in the future. Ms. X is of yet, unsure about the specifics of how she can redistribute her inheritance, but is committed to managing her own will with the help of her lawyer, and as such, has agreed to the evaluation.

### Legal Bin

The neuropsychologist in this scenario needs to consider relevant issues involved in the legal definition of testamentary capacity. The current legal standard for testamentary capacity is based in the 1870 case of *Banks* vs. *Goodfellow* (Hoffman, 2018). Generally, the legal standard for testamentary capacity is similar across states and has been fairly low, with individuals presumed to be competent unless it is proven that they are unable to fulfill one of four criteria (American Bar Association Commission on Law Aging and American Psychological Association, 2008; Hoffman, 2018):

(1) the understanding of what a will is and the fact that they are making or changing a will;
(2) the knowledge of one’s relationship to family members and other people whose interest may be affected by the will (“the object of one’s bounty”);
(3) an understanding of the nature of one’s personal property;
(4) a viable plan for the distribution of one’s property after death
  (Hoffman, 2018, p. 215).

Thus, the clinical interview and cognitive assessments selected should be designed to capture these essential elements defining testamentary capacity within the legal context. Specifically, given that Ms. X is not clear on her plan for the distribution of her property at this point, the neuropsychologist should spend specific time querying surrounding criterion four. The clinician should also reference any state-specific legal standards that may be relevant, as requirements vary by state (American Bar Association Commission on Law Aging and American Psychological Association, 2008).

### Clinical Bin

Though the client in this scenario may currently be struggling to develop a “viable plan for the distribution” of her property, the neuropsychologist in this scenario would benefit from considering whether passage of time or the implementation of proper supports may improve the client’s status on this criterion (Moberg and Kniele, 2006). For example, though the client may have cognitive and emotional difficulties currently, this does not necessarily disprove her testamentary capacity (Sousa et al., 2014; Hoffman, 2018). As referenced, the “bar” for establishing testamentary capacity is relatively low, and is task specific, varying by current level of cognitive and emotional capacity in combination with complexity of the situation (Shulman et al., 2009). Thus, this idiosyncratic capacity is not necessarily negated by the presence of neurological or psychiatric disease or even by deficits in other capacities or areas of functioning (Shulman et al., 2009). Perhaps psychotherapy to help address mood and grief as well as environmental supports to help the client better understand possible options for distribution of her wealth and assets may provide a context in which the client demonstrates adequate capacity to make these decisions (American Bar Association Commission on Law Aging and American Psychological Association, 2008). As such, it may be important to assess the client again after implementing some of these supports. Much literature to date underscores the importance of contemporaneous assessment of capacity (i.e., evaluations that occur as close to the execution of the will as possible; Shulman et al., 2009; Brenkel et al., 2018; Voskou et al., 2018), as an individual’s capacity is likely fluid rather than fixed and may be impacted by contextual changes or the implementation of environmental supports (e.g., an individual could lack capacity at one time point and possess capacity at a later time point; Moberg and Kniele, 2006). Indeed, the World Psychiatric Association’s statement on ethics and capacity in older adults with mental disorders (including dementia) emphasizes that “good clinical practice requires a flexible and supportive approach in order to optimize capacity” (Katona et al., 2009).

Additionally, given that the client has an existing baseline neuropsychological evaluation and was previously diagnosed with MCI, the neuropsychologist should review and potentially replicate assessments given at the previous evaluation in order to assess for changes over time. The neuropsychologist should keep in mind that a diagnosis of MCI (and even of a dementia) does not negate testamentary capacity given that capacity is task-specific and varies based on current cognitive and emotional functioning and complexity of the task (Shulman et al., 2009).

Finally, given the client’s cultural context, the neuropsychologist should become familiar with grief processes within the client’s religion and culture of origin. In addition, the clinician should explore the client’s values and belief system in detail in order to better understand her cultural perspective (Moberg and Kniele, 2006). Without inquiry and exploration of religious and cultural factors and values, the clinician may fall prey to confusing competency in the decision-making process with differences in value systems and the subsequent resultant will, and may thus have difficulty providing an objective assessment (Moberg and Kniele, 2006; Hoffman, 2018). The cultural context is important to consider when offering or recommending psychotherapy or psychiatric consultation as well.

### Ethical Bin

Though the ethical bin likely overlaps significantly with the other bins in this case, one specific area in the APA Ethics Code that the neuropsychologist would benefit from considering would be Standard 9, “Assessment.” Specifically, the psychologist should take care to obtain proper informed consent (Standard 9.03; American Psychological Association, 2017). The practitioner should be certain to provide a full explanation of the nature and purpose of the assessment, fees, the involvement of the client’s attorney, and any limits to confidentiality in the future given the legal context (American Psychological Association, 2017). The client should also be provided the opportunity to ask clarifying questions (American Psychological Association, 2017). Given that the evaluation is not currently court-ordered, the client should be informed that she has the right to decline the assessment, should she choose. Additionally, the clinician should keep in mind the ethical standards of selecting tests appropriate for assessment of the core functional capacities outlined in the legal definition of testamentary capacity. Hoffman (2018, p. 228) suggests that: “Cognitive testing should cover: (a) attention and concentration; (b) information processing, including receptive and expressive language; ability to understand and appreciate quantities; and executive functions such as planning; (c) and abstract and logical reasoning.” Moreover, if English is not the client’s primary language, the neuropsychologist must consider whether this may impact the overall appropriateness of the assessment (considering one’s own competency), assessment selection, and/or assessment interpretation.

Finally, given the client’s desire to be the testator of her own will, the neuropsychologist must balance the client’s rights to autonomy and self-determination (Principle E; American Psychological Association, 2017) with the possibility that if she does not have the capacity to make such financial decisions, doing so could lead to harm of the client and/or her family (Principle A; American Psychological Association, 2017). Relatedly, when considering these principles, the neuropsychologist needs to be clear about where their primary accountability lies in order to avoid a conflict of interest due to multiple relationships (Standards 3.05 and 3.06; American Psychological Association, 2017). For example, depending upon who contacted the neuropsychologist initially (e.g., client, attorney) and whether the neuropsychologist has previously worked professionally with the attorney, this multiple relationship could potentially influence the neuropsychologist’s objectivity in holding the beneficence of the client as the principal priority.

### Risk Management Bin

Though this is not currently a legal case, the neuropsychologist should keep in mind that there may be potential for further legal involvement in the future. Thus, assessing specifically for the criteria of testamentary capacity (rather than reporting on overall cognitive capacity or broad decision-making capacity) and clearly documenting how each criteria was assessed is important. The neuropsychologist should keep in mind that the court could later order that raw test data and/or the norms utilized be released, and thus this information should be precise and well-documented. Relatedly, as referenced above, the neuropsychologist should be very clear in the initial explanation to the client that, given the purpose of the evaluation, results from the assessment may very well be requested by the court, and thus, privilege may no longer apply.

## Case Vignette #2

Mr. Y is a 91-year-old, widowed, Caucasian man. His wife passed away nearly 20 years ago, and he has four children (two daughters and two sons). He is a retired farmer. Mr. Y was diagnosed with dementia with Lewy bodies approximately 2 years ago and moved to a long-term care facility shortly thereafter. He is no longer able to complete independent activities of daily living and does not operate a motor vehicle. Mr. Y’s medical providers recommended that he appoint a durable power of attorney at that point, but he refused. Mr. Y’s children report that he experiences severe anterograde memory impairment and describe marked changes in his personality over the past couple of years.

Recently, Mr. Y disinherited all of his children, deciding instead to make his former barber his primary beneficiary. Mr. Y reports that none of his children have had significant contact with him in years and that it is his desire to instead leave his inheritance to his barber, who he reports “spent more time with me over the years than any of those kids.” Mr. Y’s children are contesting his most recently executed will, claiming that he lacked testamentary capacity. The assessment is not court-ordered at this time.

### Legal Bin

Similar to the case above, the clinician should be aware of the general four criteria for testamentary capacity, as well as any specific requirements of the state. Given Mr. Y’s diagnosis, it is possible that he will struggle on standardized neuropsychological testing. This does necessarily render him incapable of testamentary capacity, however, and the neuropsychologist should thus spend ample time querying the four main criteria.

### Clinical Bin

First, given that Mr. Y lives in a long-term care facility and does not operate a motor vehicle, the neuropsychologist may want to consider offering to complete the assessment at the client’s place of residence, though privacy should be a priority (an overlap with “ethical bin;” Hoffman, 2018). Performing the evaluation in a comfortable and familiar context may help to optimize Mr. Y’s performance. Additionally, the neuropsychologist should consider any other supports or alterations to the environmental context and/or any interventions that would allow Mr. Y to demonstrate adequate capacity (Moberg and Kniele, 2006; American Bar Association Commission on Law Aging and American Psychological Association, 2008).

Given the reported changes in Mr. Y’s personality over the years, the neuropsychologist would benefit from obtaining a thorough history regarding the client’s values and preferences throughout his life (American Bar Association Commission on Law Aging and American Psychological Association, 2008). If a newly executed will is deemed a “radical departure from previously held values,” it may be deemed “unnatural” and may be an indicator of incapacity (see In re Estate of Lacy, 1967; Hoffman, 2018, p. 227). The neuropsychologist may also benefit from obtaining collateral reports from others who know Mr. Y well; his children, however, should likely be avoided given their interest in the outcome of the assessment (Hoffman, 2018). Similarly, the clinician may benefit from talking to facility staff regarding whether and how often Mr. Y’s children visit him in his current home. In addition, given that relationships may impact the choices and decisions made by older adults with mental difficulties, it would likely be important to further explore Mr. Y’s relationship with his former barber and to identify any potential for coercion or undue influence by this individual (Katona et al., 2009). The neuropsychologist should also explore any potential risk for elder abuse given Mr. Y’s potentially severe cognitive deficits (Katona et al., 2009).

Finally, the neuropsychologist should select appropriate standardized assessments to measure relevant capacities. As with the case above, it will be useful to replicate assessments that were performed at any previous evaluations in order to assess change in cognitive function over time.

### Ethical Bin

The neuropsychologist should take particular care when providing information about the informed consent process. Given that at this point, the assessment is not court-ordered, the neuropsychologist should take care to explain to Mr. Y the “nature and purpose of the evaluation,” “how the information will be used and who will have access to it,” and any potential “limitations on privacy, confidentiality, and privilege” (Hoffman, 2018, pp. 222–223). Additionally, Mr. Y should be made aware that he can refuse to participate, and he should also be informed of any potential consequences to refusal or agreement (Hoffman, 2018). Once again, the neuropsychologist should be cognizant of any potential conflicts of interest due interactions with Mr. Y’s children that could influence objectivity.

Given Mr. Y’s diagnosis, Principles A and E of the APA Ethics Code (American Psychological Association, 2017) are likely to come into prominence. A neuropsychologist has the responsibility to strive for beneficence and non-maleficence (Principle A; American Psychological Association, 2017) and to respect the rights and dignity of all people (including self-determination; Principle E; American Psychological Association, 2017), thus, this case may be primarily about balancing the client’s right to autonomy with the neuropsychologist’s duty to benefit the client and do no harm. For example, if Mr. Y’s current choices do not appear to align with his longstanding values, it is possible that changing his will now may be to his detriment, even if deeming him to lack testamentary capacity may take away some of his autonomy.

### Risk Management Bin

The case may well continue to be contested, and the neuropsychologist should keep this in mind. As noted above, the clinician should be detailed and precise in documentation, which may be even more important if Mr. Y proves incapable of participating fully in formal standardized assessment procedures. Additionally, given that this evaluation is not court-ordered at this point in time, the neuropsychologist should not solely be focused on properly obtaining informed consent, but should also be concerned with Mr. Y’s *capacity* to consent. Though Mr. Y does not currently have a durable power of attorney or a legal guardian, the neuropsychologist should keep in mind that it is possible that Mr. Y may not have the capacity to consent at all.

## Conclusion

Projections indicate that by the year 2030 approximately one in every five Americans will be an “older adult” (Colby and Ortman, 2014). Testamentary capacity litigation is already the most commonly litigated capacity (American Bar Association Commission on Law Aging and American Psychological Association, 2008; Hoffman, 2018), and older adults are involved in one of the largest transfers of wealth in history (American Bar Association Commission on Law Aging and American Psychological Association, 2008; Brenkel et al., 2018; Hoffman, 2018). Neuropsychologists are likely the most well-suited professionals to perform evaluations of testamentary capacity given their competencies and scope of practice. Though guidelines for performing these evaluations exist, there are no formal standardized evaluation procedures specifically used for determining testamentary capacity. This makes a neuropsychologist’s awareness of and attention to ethical considerations within this context ever-more important. This article has proposed that the existing four-bin model (Behnke, 2014) for approaching ethical dilemmas may be a useful approach for clinicians performing these evaluations at the clinical-legal interface. As demonstrated in the case vignettes provided above, the model may be a useful framework for organizing questions and issues involved in these cases into the most relevant categories (e.g., legal, clinical, ethical, risk management) and understanding the overlap between categories. This four-bin organization may be a useful way for neuropsychologists to move forward in determining testamentary capacity with a strong commitment to ethical practice.

## Author’s Note

This note references the hypothetical nature of the cases. The case vignettes outlined above are hypothetical and fictive in nature and are not based on any specific clinical cases.

## Author Contributions

The author confirms being the sole contributor of this work and has approved it for publication.

## Conflict of Interest Statement

The author declares that the research was conducted in the absence of any commercial or financial relationships that could be construed as a potential conflict of interest.
